# Author Correction: scCircle-seq unveils the diversity and complexity of extrachromosomal circular DNAs in single cells

**DOI:** 10.1038/s41467-024-47535-7

**Published:** 2024-04-22

**Authors:** Jinxin Phaedo Chen, Constantin Diekmann, Honggui Wu, Chong Chen, Giulia Della Chiara, Enrico Berrino, Konstantinos L. Georgiadis, Britta A. M. Bouwman, Mohit Virdi, Luuk Harbers, Sara Erika Bellomo, Caterina Marchiò, Magda Bienko, Nicola Crosetto

**Affiliations:** 1 Department of Microbiology, Tumor and Cell Biology, Karolinska Institutet, Stockholm, 17177 Sweden; 2 Science for Life Laboratory, Tomtebodavägen 23A, Solna, 17165 Sweden; 3 Biomedical Pioneering Innovation Center (BIOPIC), Peking University, Beijing, PR China; 4 School of Life Sciences, Peking University, Beijing, PR China; 5 State Key Laboratory of Biotherapy, West China Hospital, Sichuan University, Chengdu, 610041 Sichuan PR China; 6 Human Technopole, Viale Rita Levi-Montalcini 1, 22157 Milan, Italy; 7 Candiolo Cancer Institute, FPO – IRCCS, Candiolo, SP142, km 3,95, 10060 Turin, Italy; 8 Department of Medical Sciences, University of Turin, Turin, Italy; 9 Department of Oncology and Pathology, Karolinska Institutet, Stockholm, 17177 Sweden

**Keywords:** DNA sequencing, Genomic analysis, Chromosomes, Sequencing

Correction to: *Nature Communications* 10.1038/s41467-024-45972-y, published online 27 February 2024

In this article the funding line ‘Mohit Virdi is a PhD Student supported by the EURÊKA Foundation’ was omitted. The original article has been corrected.

