# Supplementary material for: scCircle-seq unveils the diversity and complexity of extrachromosomal circular DNAs in single cells
Source: Nat Commun. 2024 Feb 27;15:1768. doi: 10.1038/s41467-024-45972-y (PMC10897160; doi:10.1038/s41467-024-45972-y)
Supplement: Supplementary file 1 — Supplementary Information [file 41467_2024_45972_MOESM1_ESM.pdf]

SUPPLEMENTARY INFORMATION

scCircle-seq unveils the diversity and complexity of extrachromosomal circular DNAs in single cells

Jinxin Phaedo Chen, Constantin Diekmann, Honggui Wu, Chong Chen, Giulia Della Chiara, Enrico Berrino, Konstantinos L. Georgiadis, Britta A. M. Bouwman, Mohit Virdi, Luuk Harbers, Sara Erika Bellomo, Caterina Marchiò, Magda Bienko & Nicola Crosetto

1. Supplementary Figures pg. 2
2. Supplementary Tables pg. 22
3. Supplementary References pg. 23

1. Supplementary Figures

Supplementary Figure 1

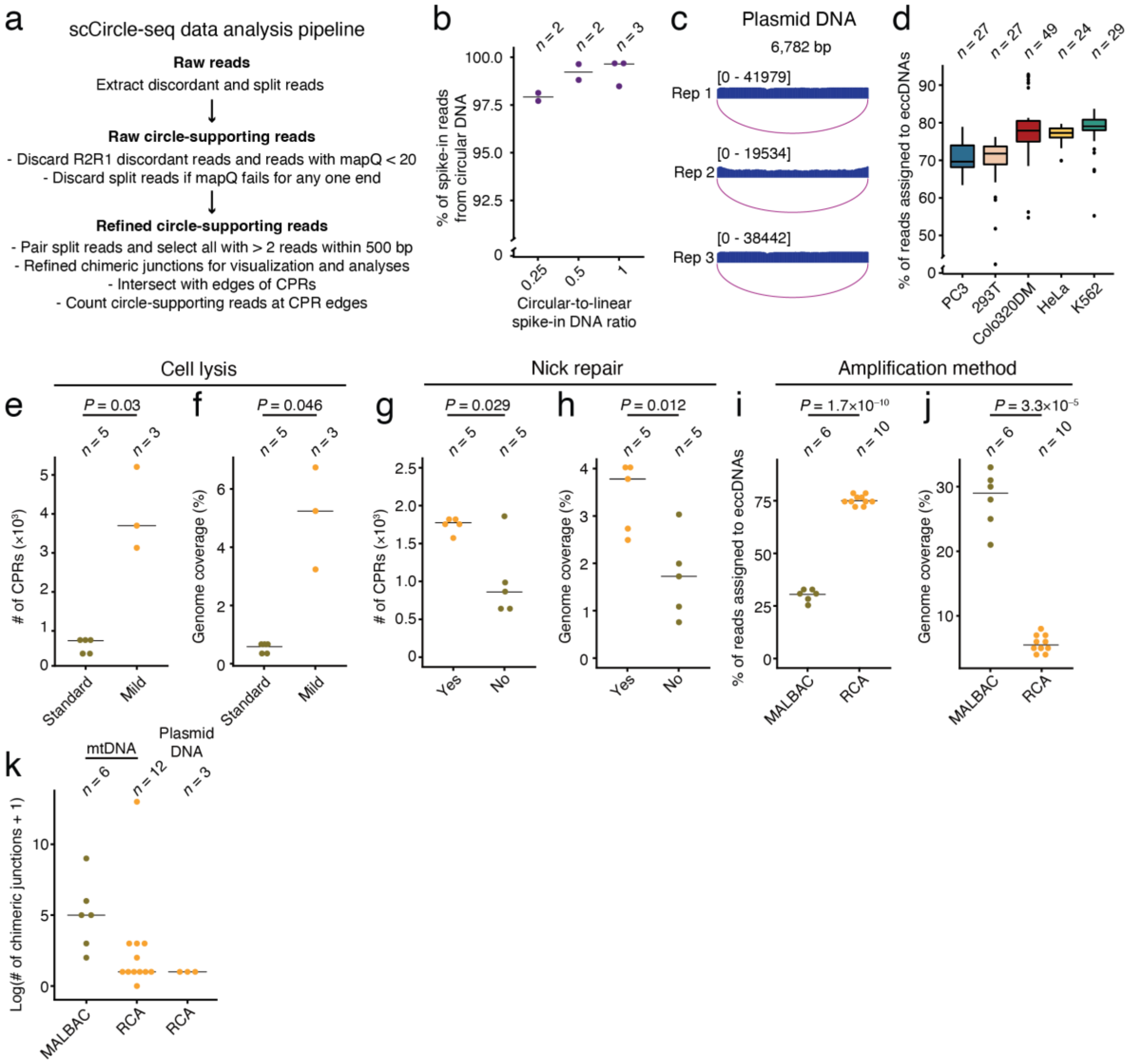

Supplementary Fig. 1. scCircle-seq implementation and optimization. (**a**) Scheme of the computational pipeline used for processing scCircle-seq data and calling circle-producing regions (CPRs). bp, base-pair. (**b**) Efficiency of linear genomic DNA (gDNA) removal during scCircle-seq, assessed by preparing mixes of different ratios of circular and linear DNA as input for scCircle-seq. *n*, number of samples. Each dot represents one sample. (**c**) Coverage (blue) and chimeric junctions identified (magenta arches) in three replicate (Rep) scCircle-seq experiments on circular plasmid DNA. The numbers in squared brackets represent the intensity range of the corresponding track. (**d**) Percentage of all reads classified as reads from eccDNAs for each of the cell lines subjected to scCircle-seq. *n*, number of single cells analyzed. Boxplots

extend from the 25th to the 75th percentile, horizontal bars represent the median, and whiskers extend from –1.5×IQR to +1.5×IQR from the closest quartile, where IQR is the inter-quartile range. Black dots, outliers. In each boxplot, the minimum and maximum are defined, respectively, by the uppermost and lowermost outlier dot or extremity of the corresponding whisker. (**e**) Number of CPRs identified by scCircle-seq in PC3 cells lysed as in Circle-Seq (standard) or using milder lysis as in scCircle-seq. *n*, number of single cells (colored dots). *P*, t-test, two-tailed. (**f**) Same as in (f) but showing the genome coverage of CPRs. (**g, h**) Same as in (e) and (f), respectively, but comparing the inclusion of nick repair in scCircle-seq (Yes) or not, as in Circle-Seq (No). (**i**) Fraction of all sequencing reads classified as reads from eccDNAs in standard scCircle-seq using rolling circle amplification (RCA) or in modified scCircle-seq using DNA amplification by multiple annealing and looping based amplification cycles (MALBAC[1]). *n*, number of single cells (colored dots). *P*, t-test, two-tailed. (**j**) Genome coverage of CPRs identified by scCircle-seq using RCA or MALBAC. *n*, number of single cells (colored dots). *P*, t-test, two-tailed. (**k**) Number of chimeric junctions detected by scCircle-seq for circular mitochondrial DNA (mtDNA) and circular plasmid DNA using two different amplification methods. *n*, number of single cells (colored dots). Source data are provided as a Source Data file.

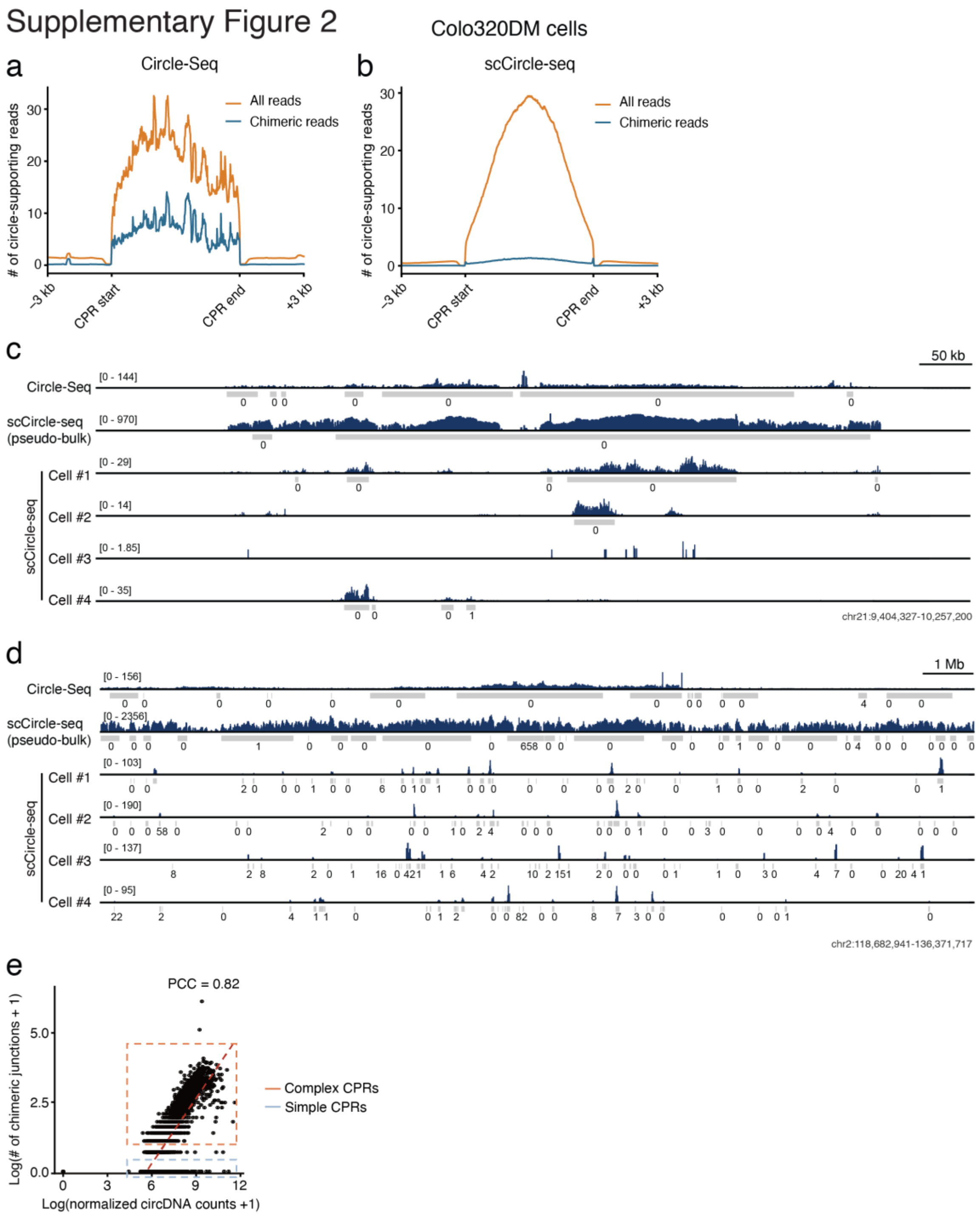

Supplementary Fig. 2. Comparison between bulk Circle-Seq and scCircle-seq. (**a, b**) Read coverage along the circle-producing regions (CPRs) identified by Circle-Seq (a) and scCircle-seq (b), separately for all reads and circle-supporting reads. kb, kilobase. (**c, d**) Integrative Genomics Viewer (IGV) tracks showing the coverage (dark blue) and called CPRs (gray) separately for bulk Circle-Seq and for four cells profiled by scCircle-seq. The numbers in squared brackets represent the intensity range of the corresponding track. The numbers below

each CPR (gray bars) indicate the number of chimeric junction-supporting reads identified in the same CPR. kb, kilobase. (**e**) Correlation between the number of eccDNAs and chimeric junctions in the CPRs detected by scCircle-seq. PCC, Pearson's correlation coefficient. Each black dot in the scatterplot represents a CPR. Dashed red line, linear regression. Complex and simple CPRs are enclosed in the dashed rectangles. Source data are provided as a Source Data file.

Supplementary Figure 3

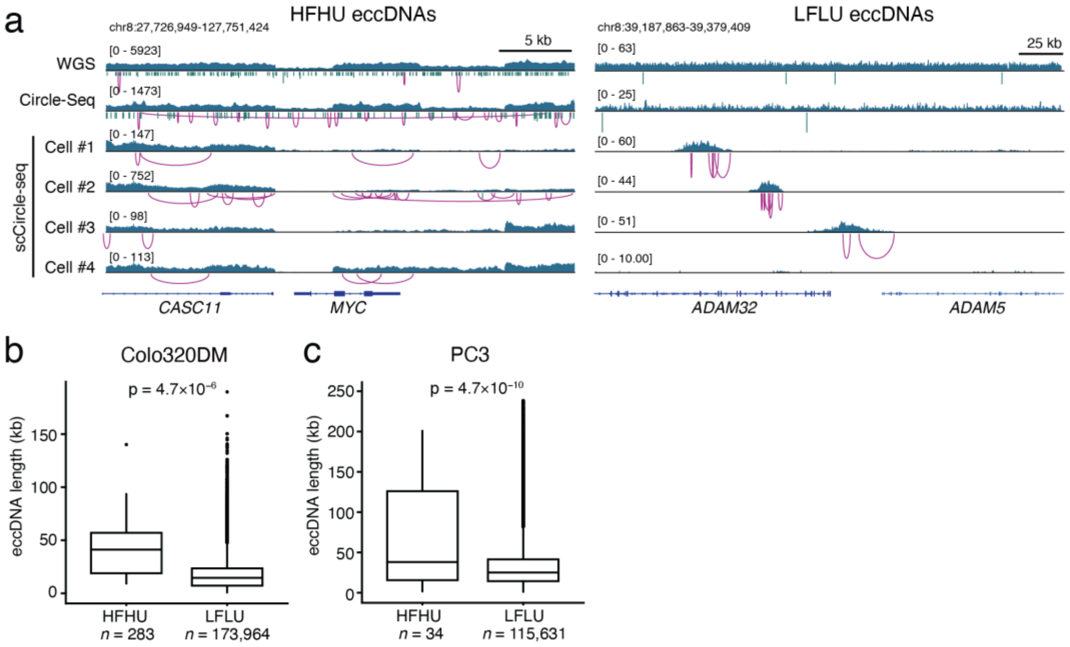

Supplementary Fig. 3. scCircle-seq detects different types of eccDNAs. (**a**) Integrative Genomics Viewer (IGV) tracks showing the coverage (dark blue) and chimeric reads (magenta arches) obtained by whole genome sequencing (WGS), Circle-Seq, and scCircle-seq, for one high-frequency high-uniformity (HFHU) eccDNA on chromosome (chr) 8 and one low-frequency low-uniformity (LFLU) eccDNA identified on the same chromosome, in Colo320DM cells. The numbers in squared brackets represent the intensity range of the corresponding track. Protein-coding genes overlapping with the regions are shown at the bottom. kb, kilobase. (**b, c**) Distributions of the lengths of the circle-producing regions associated with HFHU and LFLU eccDNAs detected by scCircle-seq in Colo320DM (b) and PC3 (c) cells. *n*, number of CPRs. *P*, t-test, two-tailed. Boxplots extend from the 25th to the 75th percentile, horizontal bars represent the median, and whiskers extend from –1.5×IQR to +1.5×IQR from the closest quartile, where IQR is the inter-quartile range. Black dots, outliers. In each boxplot, the minimum and maximum are defined, respectively, by the uppermost and lowermost outlier dot or the extremity of the corresponding whisker. Source data are provided as a Source Data file.

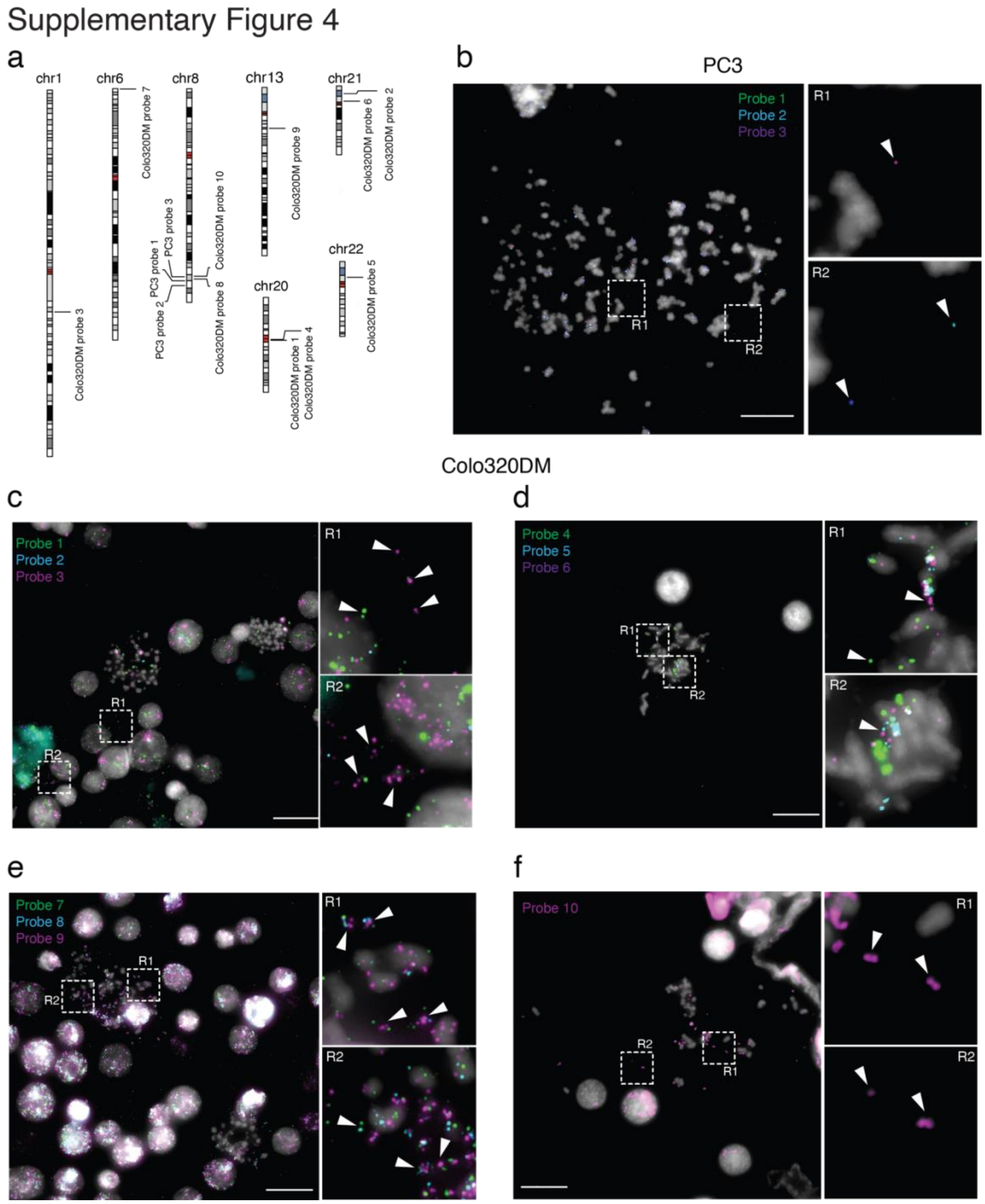

Supplementary Fig. 4. Validation of scCircle-seq by DNA fluorescence in situ hybridization (FISH). (**a**) Schematic map of the genomic location of DNA FISH probes used to validate 10 different eccDNAs detected by scCircle-seq in Colo320DM cells and 3 eccDNAs detected in PC3 cells. Probes were designed and produced using the iFISH pipeline that we previously developed[2]. (**b**) Maximum z-projection of one field of view (left image) containing two metaphase spreads of PC3 hybridized with the three probes targeting three eccDNAs detected by scCircle-seq on chromosome (chr) 8 as shown in (a). The small images on the right are magnifications of the regions (R) delimited by white dashed squares in the large image on the

left. White arrowheads indicate three eccDNA molecules that clearly reside outside of the metaphase chromosomes. Gray, DNA stained with Hoechst 33342. Scale bar: 20 μm. (**c-f**) Same as in (b) but for the ten probes targeting different eccDNAs detected in Colo320DM cells as shown in (a). Several examples of eccDNA signals overlapping with metaphase chromosomes are visible (c-e), likely reflecting the integration of these eccDNAs back into gDNA, as previously demonstrated in Colo320DM cells[3].

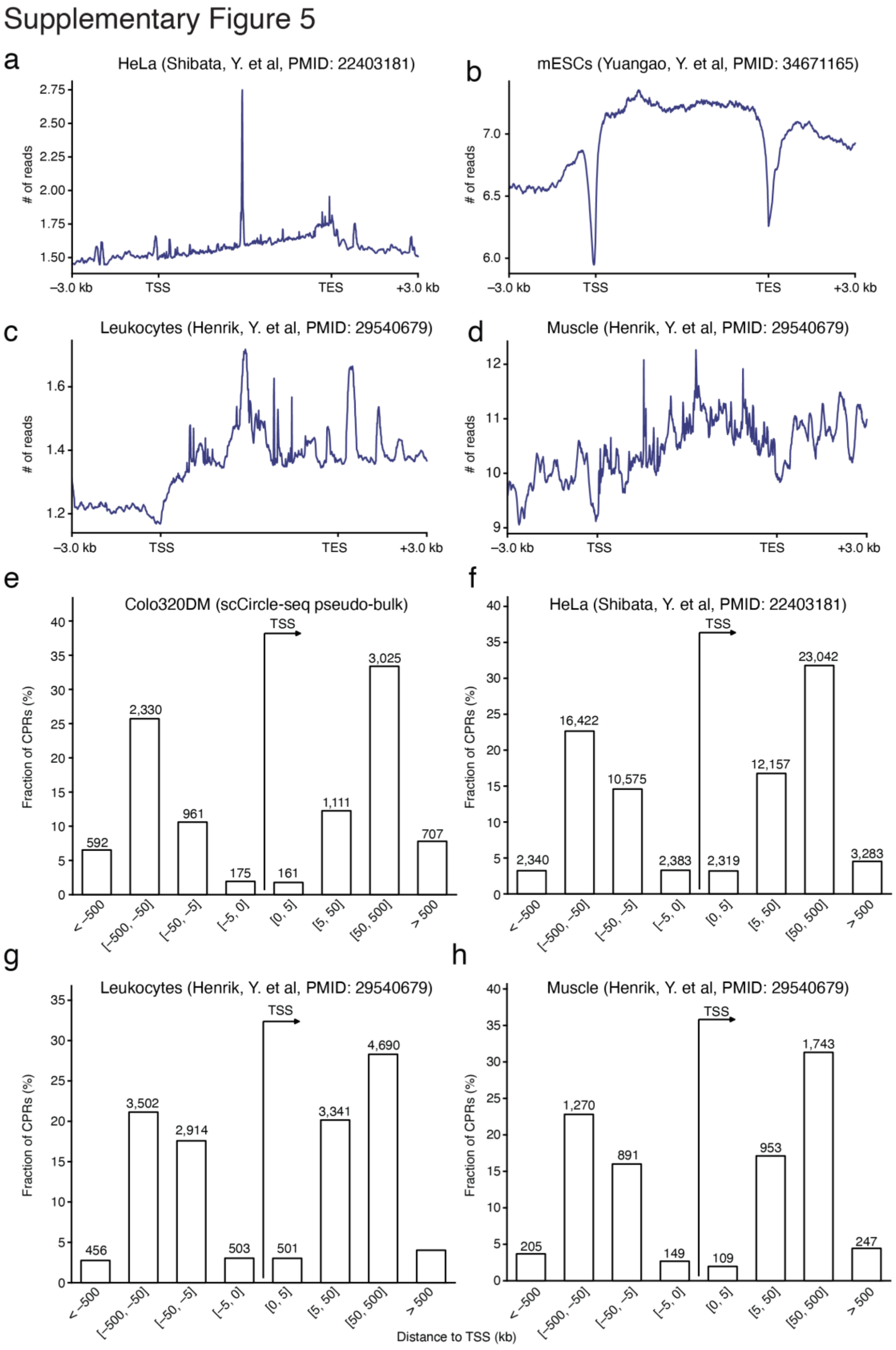

Supplementary Fig. 5. Distribution of eccDNAs along protein-coding genes. (**a-d**) Circle-Seq read coverage along the gene body of protein-coding genes in four different datasets from three

published studies. TSS, transcription start site. TES, transcription end site. PMID, PubMed identifier. kb, kilobase. *n*, number of genes. (**e-g**) Distribution of eccDNAs around the TSS of protein-coding genes, in one scCircle-seq ensemble dataset and in three published Circle-Seq datasets. The numbers on top of each bar are absolute circle-producing region (CPR) counts in the corresponding genomic interval.

Supplementary Figure 6

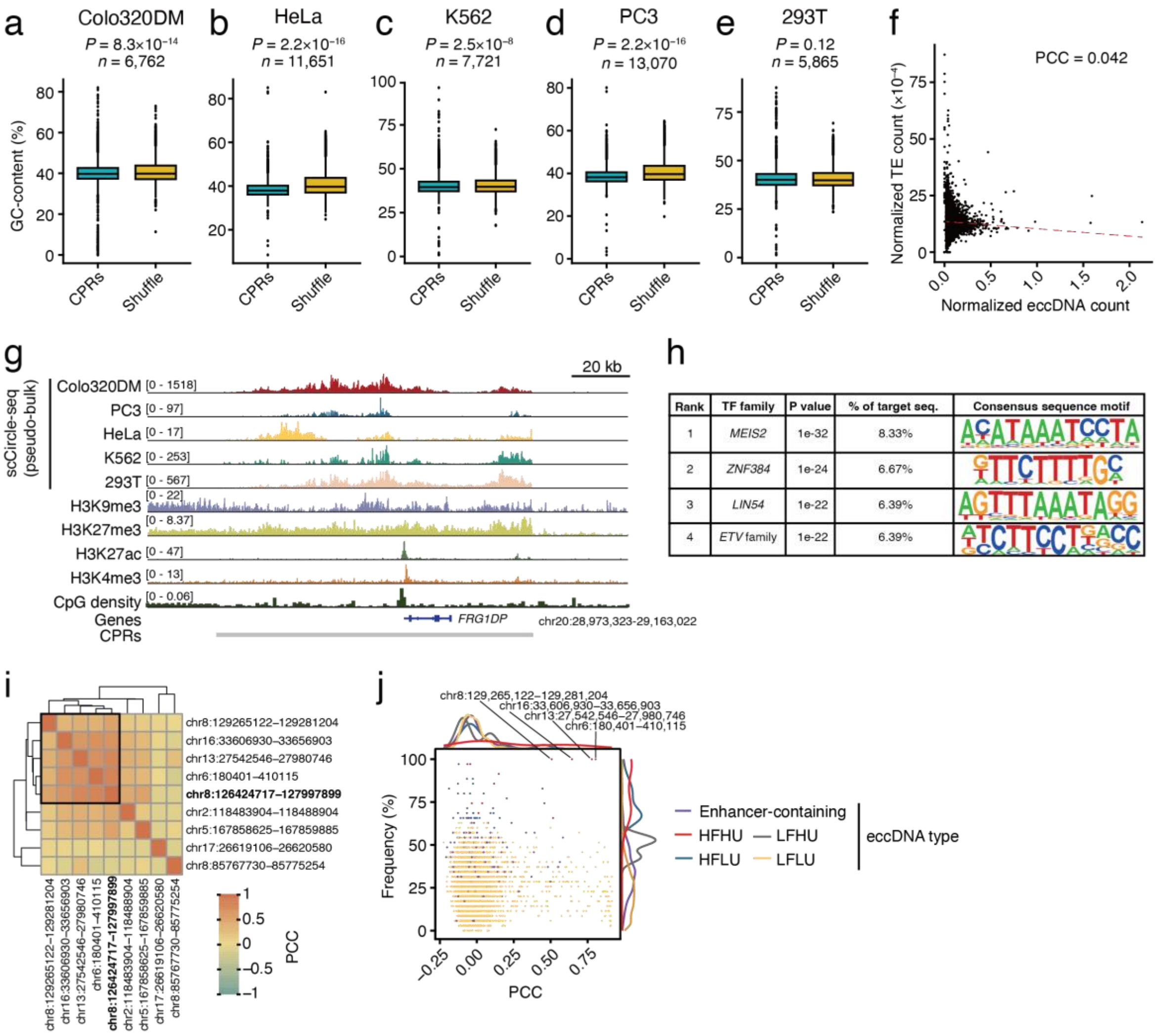

Supplementary Fig. 6. Genomic distribution of eccDNAs identified by scCircle-seq. (**a-e**) Distributions of the GC-content of circle-producing regions (CPRs) versus the same number of regions after random permutation of their genomic coordinates (shuffle), for each cell line profiled by scCircle-seq. *n*, number of CPRs. *P*, t-test, two-tailed. Boxplots extend from the 25th to the 75th percentile, horizontal bars represent the median, and whiskers extend from –1.5×IQR to +1.5×IQR from the closest quartile, where IQR is the inter-quartile range. Black dots, outliers. In each boxplot, the minimum and maximum are defined, respectively, by the uppermost and lowermost outlier dot or extremity of the corresponding whisker. (**f**) Correlation between normalized transposon element (TE) counts and normalized eccDNA counts inside the CPRs identified by scCircle-seq in HeLa cells. Each dot represents a CPR. PCC, Pearson's correlation coefficient. Dashed red line, linear regression. (**g**) Integrative Genomics Viewer (IGV) tracks showing the coverage of CPRs detected in the five cell lines profiled by scCircle-

seq and various histone marks profiled by chromatin immunoprecipitation and sequencing (ChIP-seq) in HeLa cells, in the indicated region on chromosome (chr) 20. The numbers in squared brackets represent the intensity range of the corresponding track. (**h**) Transcription factor motif enrichment inside enhancers overlapping with CPRs in Colo320DM cells. Only significantly enriched motifs are shown. (**i**) Heatmap representation of the co-occurrence of five different HFHU eccDNAs (black square) in Colo320DM cells. The region in bold corresponds to a large eccDNA (ecDNA) encompassing the *MYC* oncogene previously detected in the same cell line[4]. PCC, Pearson's correlation coefficient. (**j**) Frequency versus correlation (PCC) between the corresponding CPR and the MYC-containing ecDNA described in (i), for each CPR detected by scCircle-seq in Colo320DM cells. Each dot represents a CPR. The genomic coordinates of the four CPRs frequently co-occurring with the *MYC* ecDNA shown in (i) are displayed. Marginal distributions are shown on the top and right side of the scatterplot. Source data are provided as a Source Data file.

Supplementary Figure 7

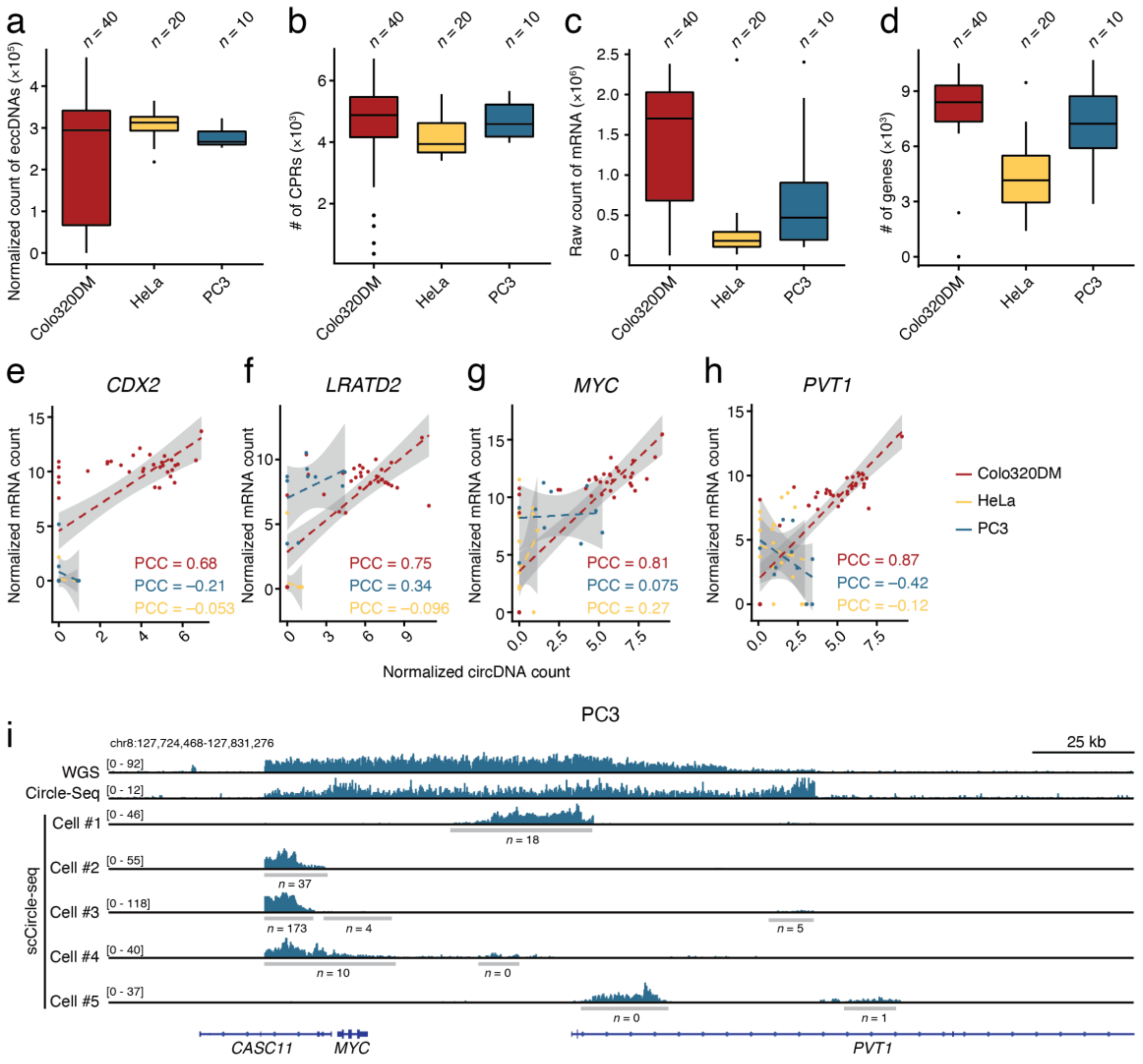

Supplementary Fig. 7. Simultaneous profiling of eccDNAs and RNA in the same cell. (**a, b**) Distributions of the normalized copy number of eccDNAs (a) and circle-producing regions (CPRs) (b) detected by scCircle-seq in three cell lines profiled by simultaneous scCircle-seq and scRNA-seq by Smart-seq2[26]. *n*, number of single cells analyzed. (**c, d**) Distributions of the raw count numbers of RNAs (c) and genes (d) detected by Smart-seq2 in the same cells shown in (a, b). In (a-d), the boxplots extend from the 25th to the 75th percentile, horizontal bars represent the median, and whiskers extend from –1.5×IQR to +1.5×IQR from the closest quartile, where IQR is the inter-quartile range. Black dots, outliers. In each boxplot, the minimum and maximum are defined, respectively, by the uppermost and lowermost outlier dot or extremity of the corresponding whisker. (**e-h**) Correlations between the normalized eccDNA counts and the mRNA counts of genes from the corresponding CPRs in each of the three cell

lines profiled by simultaneous scCircle-seq and Smart-seq2. Each dot represents one cell. Only the four genes for which the correlation was significant in at least one cell line are shown. PCC, Pearson's correlation coefficient. Dashed lines, linear regression. Gray shades, 95% confidence bands. (**i**) Integrative Genomics Viewer (IGV) tracks showing the coverage (dark blue) by whole genome sequencing (WGS), Circle-Seq, and scCircle-seq of the indicated region on chromosome (chr) 8 encompassing the *MYC* oncogene, in Colo320DM cells profiled by simultaneous scCircle-seq and Smart-seq2. Gray bars, CPRs. *n*, number of chimeric junction-supporting reads for the corresponding CPR. The numbers in squared brackets represent the intensity range of the corresponding track. Source data are provided as a Source Data file.

Supplementary Figure 8

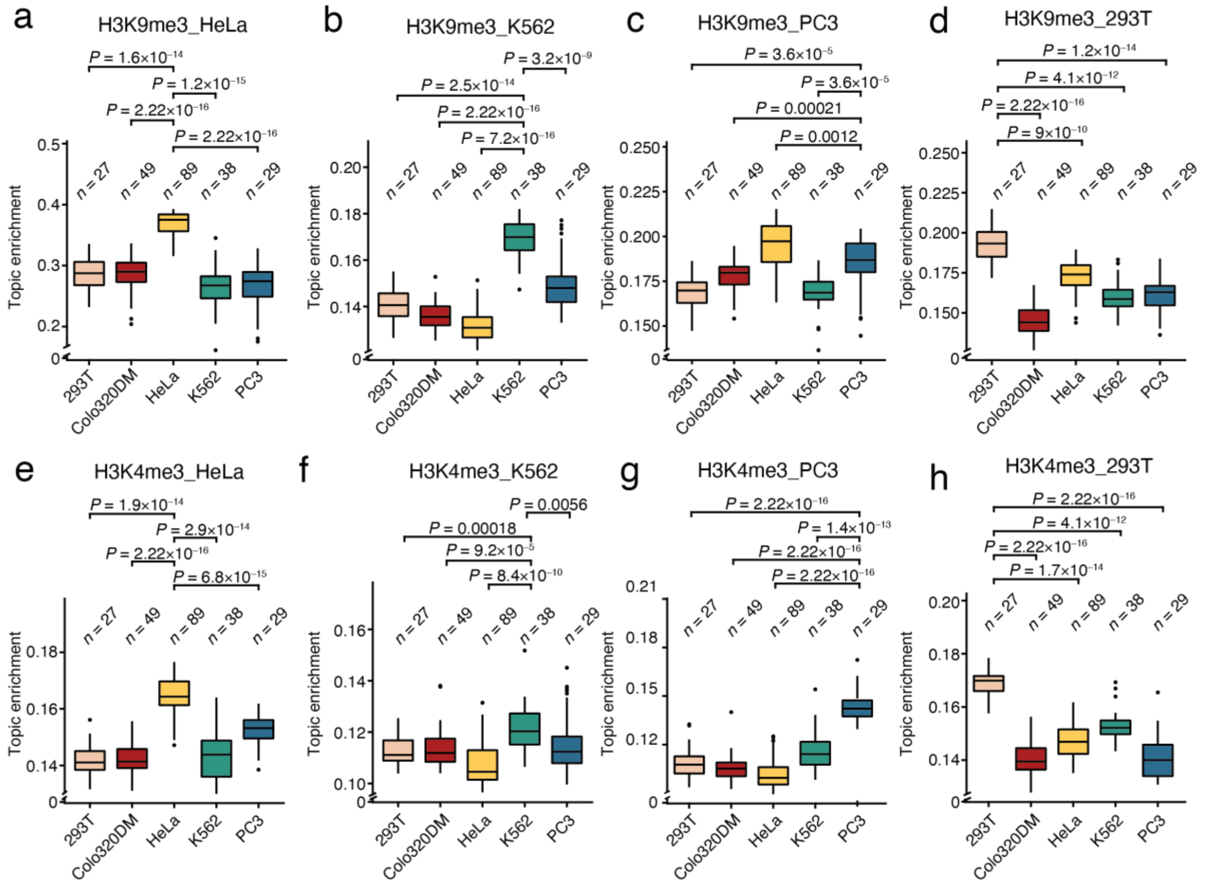

Supplementary Fig. 8. Enrichment of topics identified by cisTopic[5] related to eccDNAs in different chromatin types. (**a-d**) Topic enrichment in histone H3K9me3 chromatin immunoprecipitation and sequencing (ChIP-seq) peaks available in the Encyclopedia of DNA Elements (ENCODE) for four of the five cell lines profiled by scCircle-seq. *P*, t-test, two-tailed. (**e-h**) Same as in (a-d) but for histone H3K4me3 peaks. All boxplots in the figure extend from the 25th to the 75th percentile, horizontal bars represent the median, and whiskers extend from –1.5×IQR to +1.5×IQR from the closest quartile, where IQR is the inter-quartile range. Black dots, outliers. In each boxplot, the minimum and maximum are defined, respectively, by the uppermost and lowermost outlier dot or extremity of the corresponding whisker.

Supplementary Figure 9

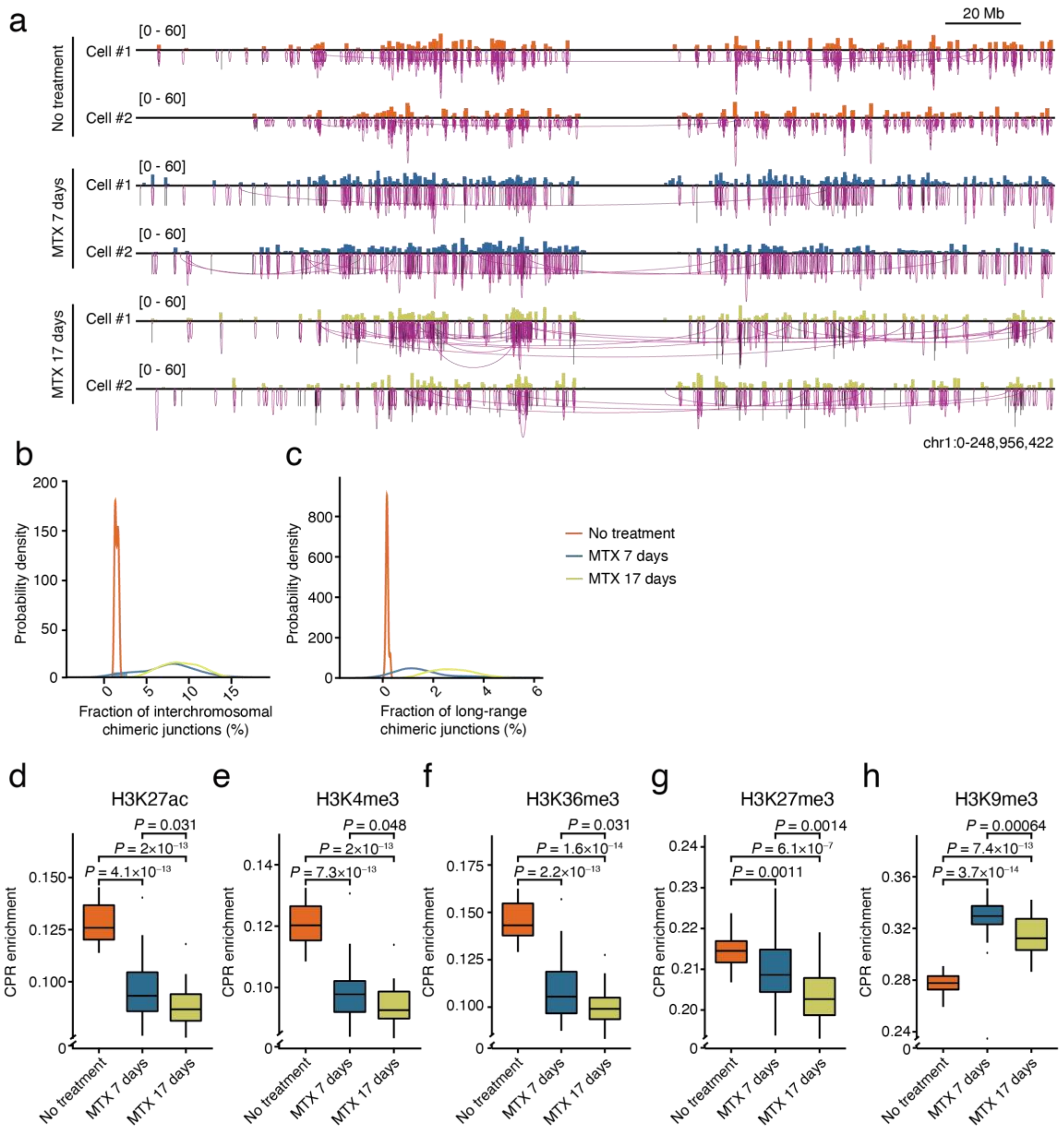

Supplementary Fig. 9. Rewiring of the eccDNA landscape upon replication stress. (**a**) Integrative Genomics Viewer (IGV) tracks showing the coverage (orange, blue, and green) and chimeric reads (magenta arches) of eccDNAs detected by scCircle-seq in HeLa cells treated or not with methotrexate (MTX). Only eccDNAs originating from chromosome (chr) 1 are shown as example. The numbers in squared brackets represent the intensity range of the corresponding track. For simplicity, only two cells per condition are shown. (**b, c**) Probability density distribution of the frequency of eccDNA chimeric junctions connecting reads that align to sequences on different chromosomes or far apart (> 100 kilobases) along the same chromosome, for HeLa cells treated or not with MTX. *n*, total number of CPRs. (**d-h**)

Enrichment of circle-producing regions (CPRs) inside chromatin immunoprecipitation and sequencing (ChIP-seq) peaks for the indicated histone marks available in the Encyclopedia of DNA Elements (ENCODE), for HeLa cells treated or not with MTX. *P*, t-test, two-tailed. Source data are provided as a Source Data file.

Supplementary Figure 10

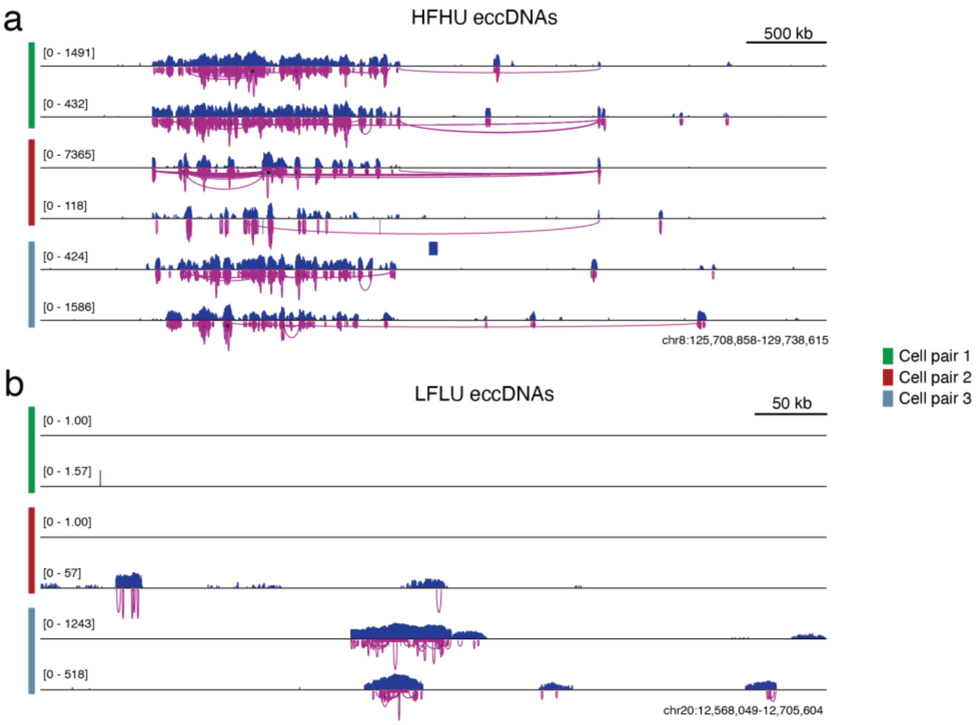

Supplementary Fig. 10. Divergence of eccDNAs between daughter cells after mitosis. (**a**) Integrative Genomics Viewer (IGV) tracks showing the coverage (blue) and chimeric reads (magenta arches) of a high-frequency high-uniformity (HFHU) eccDNA on chromosome (chr) 8 detected by scCircle-seq in three pairs of HeLa daughter cells picked immediately after mitosis. The numbers in squared brackets represent the intensity range of the corresponding track. (**b**) Same as in (a) but for a low-frequency low-uniformity (LFLU) eccDNA on chr 20.

Supplementary Figure 11

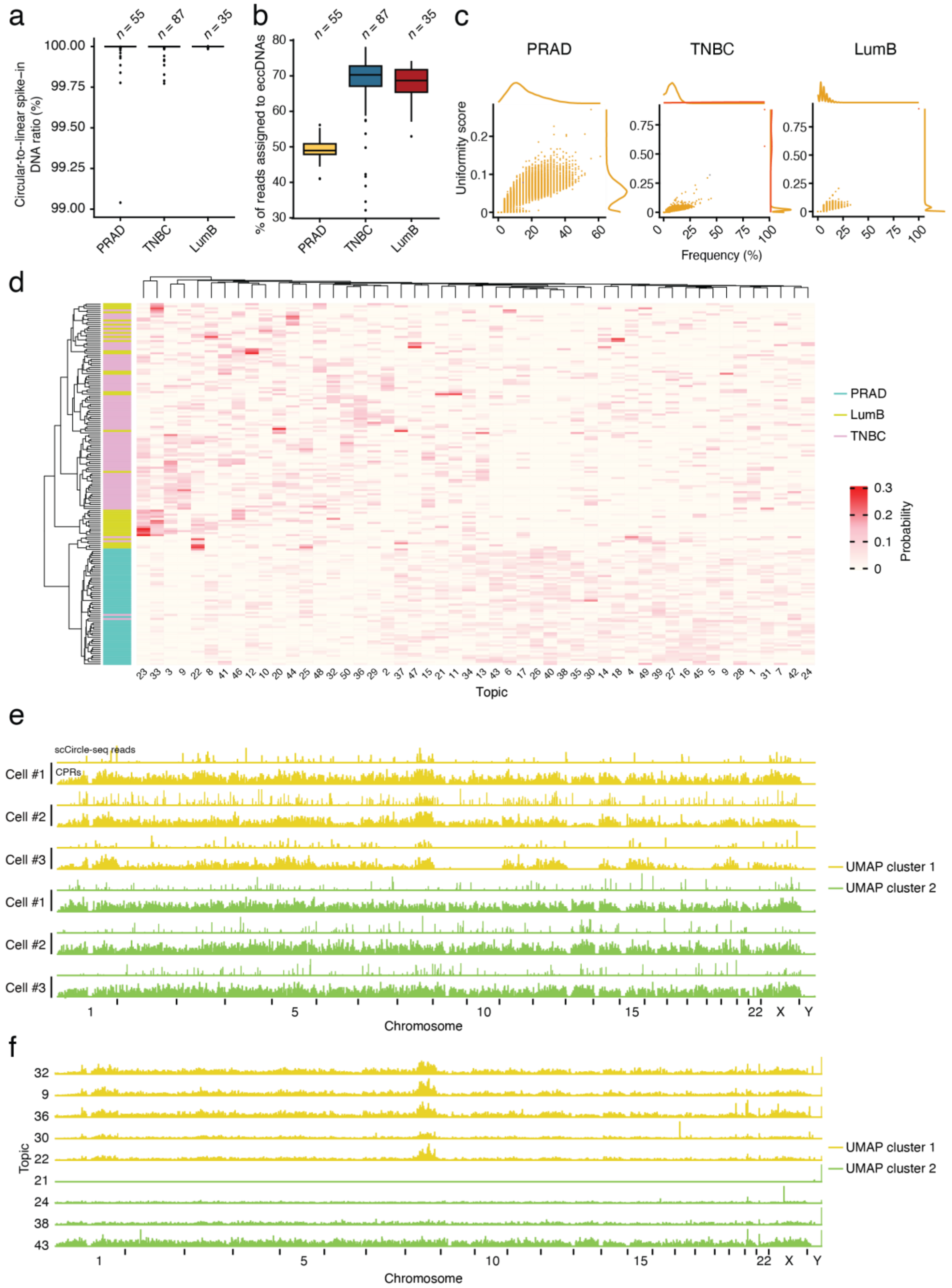

Supplementary Fig. 11. Proof-of-principle application of scCircle-seq to patient-derived tumor samples. (**a**) Distributions of the ratio between circular and linear spike-in DNA in three

independent scCircle-seq experiments performed on nuclei isolated from one prostate adenocarcinoma (PRAD), one Luminal B-like breast cancer (LumB), and one triple-negative breast cancer (TNBC). *n*, number of single cells analyzed. (**b**) Percentage of all the sequencing reads assigned to eccDNAs for the same samples shown in (a). *n*, number of single cells analyzed. In (a) and (b), boxplots extend from the 25th to the 75th percentile, horizontal bars represent the median, and whiskers extend from –1.5×IQR to +1.5×IQR from the closest quartile, where IQR is the inter-quartile range. Black dots, outliers. In each boxplot, the minimum and maximum are defined, respectively, by the uppermost and lowermost outlier dot or extremity of the corresponding whisker. (**c**) Correlation between the frequency and coverage of the four different types of eccDNAs schematically shown in **Fig. 2d**, for each of the three tumor samples profiled by scCircle-seq. Colors are the same as in **Fig. 2d**. Each dot in the scatterplots represents a circle-producing region (CPR). Marginal distributions are shown on the top and right side of each scatterplot. Essentially, only low frequency low uniformity (LFLH) eccDNAs were detected in these tumor samples. (**d**) Heatmap representation of topic contribution for 177 nuclei extracted from tumor samples and profiled by scCircle-seq. Cells and topics are clustered hierarchically. Cells are colored by cell type as indicated. Each row represents one cell. (**e**) scCircle-seq read coverage (bottom track) and CPR distribution (upper track) for three TNBC cells assigned to UMAP Cluster-1 and three cells assigned to Cluster-2. See **Fig. 4f** for the corresponding UMAP. (**f**) Genome-wide contribution to the top-5 enriched topics in TNBC Cluster-1 (yellow) or Cluster-2 (green) cells. A strong contribution to the topics enriched in Cluster-1 comes from a region on chromosome 8 that encompasses the MYC gene and that is frequently amplified across breast cancers[6]. Source data are provided as a Source Data file.

Supplementary Figure 12

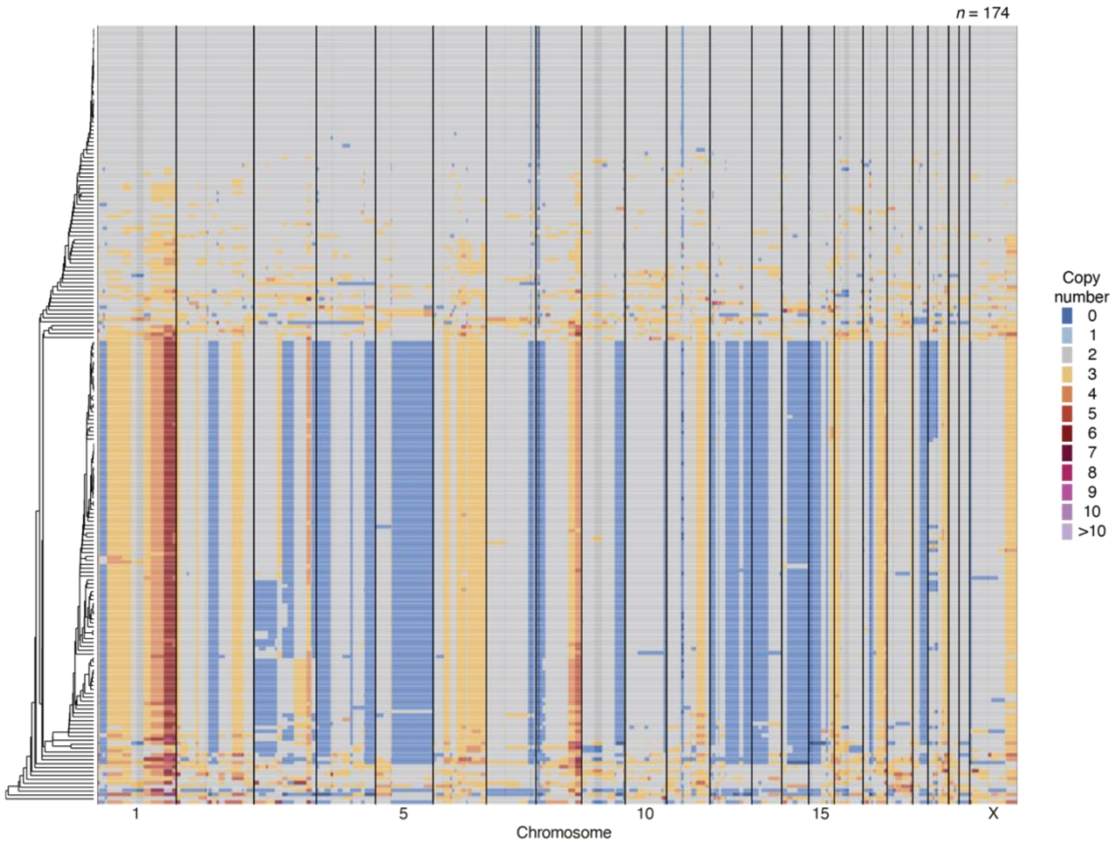

Supplementary Fig. 12. DNA copy number profiles (500 kilobase, kb resolution) of individual nuclei extracted from the same triple-negative breast cancer (TNBC) sample profiled by scCircle-seq. The nuclei were processed through Acoustic Cell Tagmentation (ACT)[7] as described in the **Methods**. Each row represents one cell. Cells are clustered hierarchically. *n*, number of cells analyzed.

2. Supplementary Tables

Supplementary Table 1. Summary of publicly available downloaded data.

Cell line	Method	Database	Accession Number
Colo320DM	Whole genome sequencing	SRA	SRX5055021
PC3	Whole genome sequencing	SRA	SRX5055020
PC3	ChIP-seq for H3K4me3	SRA	GSM3768252
PC3	ChIP-seq for H3K9me3	ENCODE	ENCSR339ZMJ
PC3	ChIP-seq for H3K27ac	SRA	GSM1383871
PC3	ChIP-seq for H3K27me3	ENCODE	ENCSR881TWJ
HeLa	ChIP-seq for H3K4me3	SRA	GSM3398461
HeLa	ChIP-seq for H3K9me3	SRA	GSM4710592
HeLa	ChIP-seq for H3K27ac	SRA	GSM2990412
HeLa	ChIP-seq for H3K27me3	SRA	GSM2990413
K562	ChIP-seq for H3K4me3	ENCODE	ENCSR000EW
K562	ChIP-seq for H3K9me3	SRA	GSM5175742
K562	ChIP-seq for H3K27ac	SRA	GSM5593404
K562	ChIP-seq for H3K27me3	ENCODE	ENCSR000AKQ
293T	ChIP-seq for H3K4me3	SRA	GSM5954235
293T	ChIP-seq for H3K9me3	ENCODE	ENCSR000FCJ
293T	ChIP-seq for H3K27ac	SRA	GSM5954237
293T	ChIP-seq for H3K27me3	SRA	GSM5269337

3. Supplementary References

1. Zong, C., Lu, S., Chapman, A. R. & Xie, X. S. Genome-wide detection of single-nucleotide and copy-number variations of a single human cell. *Science* **338**, 1622–1626 (2012).
2. Gelali, E. *et al.* iFISH is a publically available resource enabling versatile DNA FISH to study genome architecture. *Nat. Commun.* **10**, 1636 (2019).
3. Nathanson, D. A. *et al.* Targeted therapy resistance mediated by dynamic regulation of extrachromosomal mutant EGFR DNA. *Science* **343**, 72–76 (2014).
4. Hung, K. L. *et al.* ecDNA hubs drive cooperative intermolecular oncogene expression. *Nature* **600**, 731–736 (2021).
5. Bravo González-Blas, C. *et al.* cisTopic: cis-regulatory topic modeling on single-cell ATAC-seq data. *Nat. Methods* **16**, 397–400 (2019).
6. Cancer Genome Atlas Network. Comprehensive molecular portraits of human breast tumours. *Nature* **490**, 61–70 (2012).
7. Minussi, D. C. *et al.* Breast tumours maintain a reservoir of subclonal diversity during expansion. *Nature* **592**, 302–308 (2021).
