# Supplementary material for: scCircle-seq unveils the diversity and complexity of extrachromosomal circular DNAs in single cells
Source: Nat Commun. 2024 Feb 27;15:1768. doi: 10.1038/s41467-024-45972-y (PMC10897160; doi:10.1038/s41467-024-45972-y)
Supplement: Supplementary file 3 — Description of Additional Supplementary Files [file 41467_2024_45972_MOESM3_ESM.pdf]

Description of Additional Supplementary Files

Supplementary Data 1. Summary of scCircle-seq sequencing data described in this study.

Supplementary Data 2. Sequences of the oligos composing the DNA FISH probes used for scCircle-seq validation.

Supplementary Data 3. Summary of ACT sequencing data described in this study.
