# Supplementary material for: scCircle-seq unveils the diversity and complexity of extrachromosomal circular DNAs in single cells
Source: Nat Commun. 2024 Feb 27;15:1768. doi: 10.1038/s41467-024-45972-y (PMC10897160; doi:10.1038/s41467-024-45972-y)
Supplement: Supplementary file 7 — Reporting Summary [file 41467_2024_45972_MOESM7_ESM.pdf]

nature portfolio

Corresponding author(s): Nicola Crosetto

Last updated by author(s): Jan 17, 2024

Reporting Summary

Nature Portfolio wishes to improve the reproducibility of the work that we publish. This form provides structure for consistency and transparency in reporting. For further information on Nature Portfolio policies, see our Editorial Policies and the Editorial Policy Checklist.

Statistics

For all statistical analyses, confirm that the following items are present in the figure legend, table legend, main text, or Methods section.

n/a	Confirmed	
☐	☒	The exact sample size (*n*) for each experimental group/condition, given as a discrete number and unit of measurement
☒	☐	A statement on whether measurements were taken from distinct samples or whether the same sample was measured repeatedly
☐	☒	The statistical test(s) used AND whether they are one- or two-sided<br>*Only common tests should be described solely by name; describe more complex techniques in the Methods section.*
☒	☐	A description of all covariates tested
☒	☐	A description of any assumptions or corrections, such as tests of normality and adjustment for multiple comparisons
☐	☒	A full description of the statistical parameters including central tendency (e.g. means) or other basic estimates (e.g. regression coefficient) AND variation (e.g. standard deviation) or associated estimates of uncertainty (e.g. confidence intervals)
☐	☒	For null hypothesis testing, the test statistic (e.g. *F*, *t*, *r*) with confidence intervals, effect sizes, degrees of freedom and *P* value noted<br>*Give P values as exact values whenever suitable.*
☒	☐	For Bayesian analysis, information on the choice of priors and Markov chain Monte Carlo settings
☒	☐	For hierarchical and complex designs, identification of the appropriate level for tests and full reporting of outcomes
☐	☒	Estimates of effect sizes (e.g. Cohen's *d*, Pearson's *r*), indicating how they were calculated

Our web collection on statistics for biologists contains articles on many of the points above.

Software and code

Policy information about availability of computer code

Data collection

All data described in this study were collected by massively parallel DNA sequencing on Illumina platforms, using their associated proprietary software. Raw bcl2 sequencing files were converted to fastq files using Illumina's free BaseSpace cloud platform.

Data analysis

All the scripts used to process and analyze the scCircle-seq data described in this study are available at https://github.com/BiCroLab/scCircle-seq.

We used the following open source packages:
Bwa (version 0.7.17-r1188)
Samtools (version 1.6)
Picard Tools (version 2.25.5-2)
bedtools (version 2.3.0)
deepTools (version 3.5.0)
pgltools (verion 2.2.0)
cisTopic (version 3)

For manuscripts utilizing custom algorithms or software that are central to the research but not yet described in published literature, software must be made available to editors and reviewers. We strongly encourage code deposition in a community repository (e.g. GitHub). See the Nature Portfolio guidelines for submitting code & software for further information.

Data

Policy information about availability of data

All manuscripts must include a data availability statement. This statement should provide the following information, where applicable:

- Accession codes, unique identifiers, or web links for publicly available datasets
- A description of any restrictions on data availability
- For clinical datasets or third party data, please ensure that the statement adheres to our policy

All scCircle-seq data described in this study are summarized in Supplementary Table 1.
Sequencing data obtained from cell lines are publically available on the GEO database at: https://www.ncbi.nlm.nih.gov/geo/query/acc.cgi?acc=GSE221884.
Raw sequencing data from the patient samples are available on ENA database at https://www.ebi.ac.uk/ena/browser/view/PRJEB71652.
Pre-processed sequencing data from patient samples are available at https://figshare.com/account/home#/projects/183466.
Whole genome sequencing of Colo320DM, https://www.ncbi.nlm.nih.gov/sra/?term=SRX5055021
Whole genome sequencing of PC3, https://www.ncbi.nlm.nih.gov/sra/?term=SRX5055020
H3K4me3 ChIP-seq for PC3, https://www.ncbi.nlm.nih.gov/sra/?term=GSM3768252
H3K9me3 ChIP-seq for PC3, https://www.encodeproject.org/experiments/ENCSR339ZMJ/
H3K27ac ChIP-seq for PC3, https://www.ncbi.nlm.nih.gov/sra/?term=GSM1383871
H3K27me3 ChIP-seq for PC3, https://www.encodeproject.org/experiments/ENCSR881TWJ/
H3K4me3 ChIP-seq for HeLa, https://www.ncbi.nlm.nih.gov/sra/?term=GSM3398461
H3K9me3 ChIP-seq for HeLa, https://www.ncbi.nlm.nih.gov/sra/?term=GSM4710592
H3K27ac ChIP-seq for HeLa, https://www.ncbi.nlm.nih.gov/sra/?term=GSM2990412
H3K27me3 ChIP-seq for HeLa, https://www.ncbi.nlm.nih.gov/sra/?term=GSM2990413
H3K4me3 ChIP-seq for K562,https://www.encodeproject.org/experiments/ENCSR000EWA/
H3K9me3 ChIP-seq for K562, https://www.ncbi.nlm.nih.gov/sra/?term=GSM5175742
H3K27ac ChIP-seq for K562, https://www.ncbi.nlm.nih.gov/sra/?term=GSM5593404
H3K27me3 ChIP-seq for K562, https://www.encodeproject.org/experiments/ENCSR000AKQ/
H3K4me3 ChIP-seq for 293T, https://www.ncbi.nlm.nih.gov/sra/?term=GSM5954235
H3K9me3 ChIP-seq for 293T, https://www.encodeproject.org/experiments/ENCSR000FCJ/
H3K27ac ChIP-seq for 293T, https://www.ncbi.nlm.nih.gov/sra/?term=GSM5954237
H3K27me3 ChIP-seq for 293T, https://www.ncbi.nlm.nih.gov/sra/?term=GSM5269337
A summary statistics of ACT data described in this study is available in Supplementary Table 4.

Research involving human participants, their data, or biological material

Policy information about studies with human participants or human data. See also policy information about sex, gender (identity/presentation), and sexual orientation and race, ethnicity and racism.

Reporting on sex and gender	All participants for which tumor tissue was used in this project reported their sex and gender during their clinical evaluation, which was in line with the information on their identity cards. The participants involved in the prostate cancer cohort reported male sex. The participants involved in the breast cancer cohort reported female sex. We included these samples solely to demonstrate the applicability of our method to patient-derived biopsies.
Reporting on race, ethnicity, or other socially relevant groupings	All participants were European. We do, however, not utilize information on race, ethnicity, or other socially relevant grouping of the humans involved in this study for the conclusions of this study. We included these samples solely to demonstrate the applicability of our method to patient-derived biopsies.
Population characteristics	Participants in the prostate cancer cohort were 43–65 years old. For the breast cancer samples, the donors were 58 and 65 years of age at the time of sample collection. We, however, do not use the samples to relate outcome to age in any way, and solely included these samples to demonstrate applicability of our method.
Recruitment	Participants were informed of the possibility to join by their clinician after diagnosis. There was no compensation.
Ethics oversight	The permit for the prostate cancer sample was applied for by Södersjukhuset hospital in Stockholm, Sweden (permit number #2018/1003-31), for which the permit was granted by EPN, regionala etikprövningsnämden Stockholm.<br>The permit for the breast cancer samples was obtained by the Pathology Unit of the Candiolo Cancer Institute, Turin, Italy (001-IRCC-00IIS-10), which was approvded by the Ethical Committee of Fondazione Piemontese per l'Oncologia - Instituto di Ricerca e Cura a Carattere Scientifico of Candiolo.

Note that full information on the approval of the study protocol must also be provided in the manuscript.

Field-specific reporting

Please select the one below that is the best fit for your research. If you are not sure, read the appropriate sections before making your selection.

☒ Life sciences ☐ Behavioural & social sciences ☐ Ecological, evolutionary & environmental sciences

For a reference copy of the document with all sections, see nature.com/documents/nr-reporting-summary-flat.pdf

Life sciences study design

All studies must disclose on these points even when the disclosure is negative.

Sample size	We did not perform any sample size calculation as this is not relevant when setting up a new single-cell sequencing method, also because we're not comparing different treatment types between samples/patients. We only want to demonstrate that our method works.<br>We choose >=2 cells as the sample size for all the benchmark experiments.<br>We performed all the benchmark experiments in 2 or more biological replicates to capture variability.<br>We conducted the drug-treatment experiments with 10 or more cells for assessing different treatments and to capture cell heterogeneity.<br>For the paired-cell experiments, we performed 2 biological replicate experiments.
Data exclusions	For scCircle-seq data, we mapped raw reads to the hg38 reference genome and called circle producing regions (CPRs) as described in the Methods section. We filtered out cells if the mapping ratio was below 70% or the fraction of reads within CPRs was below 30%. We did not exclude any patients either, but rather used only one (prostate cancer cohort) and two (breast cancer cohort) for demonstration purposes and show applicability of the method.
Replication	We performed all experiments on cell lines in 2 or more biological replicates. And all the replicates were successful.
Randomization	We performed all the experiments described in this study on cultured immortalized cell lines or on nuclei extracted from patient tumor biopsies collected without randomization since the samples themselves are random already.
Blinding	This study did not include any blinding since it is exploratory.

Reporting for specific materials, systems and methods

We require information from authors about some types of materials, experimental systems and methods used in many studies. Here, indicate whether each material, system or method listed is relevant to your study. If you are not sure if a list item applies to your research, read the appropriate section before selecting a response.

Materials & experimental systems

n/a	Involved in the study	
☒	☐	Antibodies
☐	☒	Eukaryotic cell lines
☒	☐	Palaeontology and archaeology
☒	☐	Animals and other organisms
☒	☐	Clinical data
☒	☐	Dual use research of concern
☒	☐	Plants

Methods

n/a	Involved in the study	
☒	☐	ChIP-seq
☒	☐	Flow cytometry
☒	☐	MRI-based neuroimaging

Eukaryotic cell lines

Policy information about cell lines and Sex and Gender in Research

Cell line source(s)	We used the following cell lines purchased from ATCC: Colo320DM (cat. no. CCL-220), PCR3 (cat. no. CRL-1435), HeLa (cat. no. CCL-2), HEK293T (cat. no. CRL-1573), K562 (cat. no. CCL-243).
Authentication	We did not perform authentication of any of the cell lines used in this study.
Mycoplasma contamination	The cell lines used were test negative on Mycoplasma in our laboratory.
Commonly misidentified lines<br>(See ICLAC register)	None of the cell lines used in this study are listed in the ICLAC register.

Plants

Seed stocks	N/A
Novel plant genotypes	N/A
Authentication	N/A
